# Elevated *MPP6* expression correlates with an unfavorable prognosis, angiogenesis and immune evasion in hepatocellular carcinoma

**DOI:** 10.3389/fimmu.2023.1173848

**Published:** 2023-05-03

**Authors:** Qianqian Cheng, Wei Wang, Jing Liu, Zhenyu Lv, Wenbin Ji, Jinhui Yu, Wenting Zhang, Yan Yang

**Affiliations:** Department of Medical Oncology, The First Affiliated Hospital of Bengbu Medical College, Bengbu, China

**Keywords:** angiogenesis, hepatocellular carcinoma, immune evasion, MPP6, prognosis, treatment response

## Abstract

**Background:**

Membrane palmitoylated proteins (MPPs) are engaged in various biological processes, such as cell adhesion and cell polarity. Dysregulated MPP members have different effects on hepatocellular carcinoma (HCC) development. However, the role of *MPP6* in HCC has been unknown.

**Method:**

HCC transcriptome and clinical data from different public databases were downloaded and analyzed, and the results were further validated by qRT−PCR, Western blotting and immunohistochemistry (IHC) using HCC cell lines and tissues. The association between *MPP6* and prognosis, potential pathogenic mechanisms, angiogenesis, immune evasion, tumor mutation burden (TMB) and treatment response in HCC patients was analyzed by bioinformatics and IHC staining.

**Results:**

*MPP6* was significantly overexpressed in HCC, and its expression was related to T stage, pathologic stage, histologic grade and adverse prognosis in HCC patients. Gene set enrichment analysis revealed that differentially expressed genes were mainly enriched in the synthesis of genetic materials and the WNT signaling pathway. GEPIA database analysis and IHC staining suggested that *MPP6* expression had a positive correlation with angiogenesis. Single-cell dataset analysis indicated that *MPP6* was associated with features of the tumor microenvironment. Additional analyses discovered that *MPP6* expression was inversely related to immune cell infiltration and was involved in tumor immune evasion. *MPP6* expression was positively associated with TMB, and patients with high TMB had an adverse prognosis. Immunotherapy was more effective in HCC patients with low *MPP6* expression, whereas those with high *MPP6* expression responded better to sorafenib, gemcitabine, 5-FU, and doxorubicin.

**Conclusions:**

Elevated *MPP6* expression is associated with an unfavorable prognosis, angiogenesis and immune evasion in HCC. Moreover, *MPP6* has the potential to be used to assess TMB and treatment response. Therefore, *MPP6* might serve as a novel prognostic biomarker and therapeutic target for HCC.

## Graphical Abstract

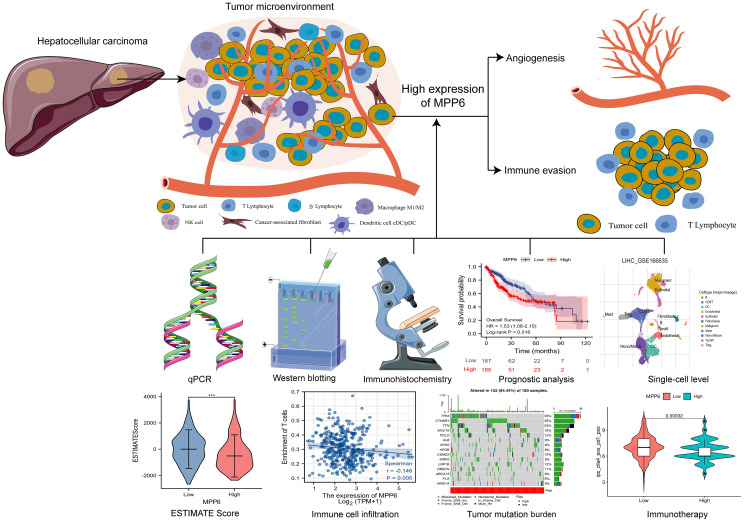

## 1 Introduction

Primary liver cancer (PLC) is one of the most prevalent malignancies worldwide (1–3). Hepatocellular carcinoma (HCC) accounts for approximately 85%-90% of PLC cases. Early diagnosis and treatment of HCC is ideal; however, many HCC patients are diagnosed at advanced stages, at which point the survival is unfavorable. Currently, the mechanism of HCC pathogenesis is unclear; hence, it is crucial to explore HCC pathological mechanisms and to recognize biomarkers that affect HCC initiation, progression and prognosis.

Membrane palmitoylated proteins (MPPs), which are mainly located at cell−cell junctions and include MPP1-MPP7, constitute an important subfamily of membrane-associated guanylate kinases (MAGUKs). MPPs can engage in various biological processes, such as cell adhesion, and cell polarity regulation (4–6), and abnormalities in MPPs functions may lead to malignant cell transformation and mediate tumor invasion and metastasis (7, 8). Although there have been great achievements in MPPs research over the years, the role of MPPs in malignant tumors is not completely understood (9–12). Li et al. (10) found that the upregulation of MPP2 promoted apoptosis and suppressed the spreading of HCC cells. However, MPP3 had increased expression in HCC tissues, which facilitated HCC cell migration and invasion and was related to unfavorable survival in a study (11). Above studies indicate that MPP members may have different effects on HCC development. MPP6 has been identified as an exosome-related RNA-binding protein (13) and has been demonstrated to inhibit ovarian cancer progression and prolong survival in ovarian cancer patients (12); however, the role of *MPP6* in HCC has been unknown. This study explored *MPP6* expression in HCC based on multiple databases and basic research and further assessed the correlations between *MPP6* expression and prognosis, potential pathogenic mechanisms, angiogenesis, immune evasion, tumor mutation burden (TMB) and treatment response in HCC patients, aiming to provide a new potential prognostic biomarker and therapeutic target for HCC.

## 2 Materials and methods

### 2.1 Data collection

HCC transcriptome and clinical data were downloaded from The Cancer Genome Atlas (TCGA) database (14) (https://portal.gdc.cancer.gov/) with assessment date in September 2022, and the analyses results were verified by Japan data released in International Cancer Genome Consortium (ICGC) database (15) (https://dcc.icgc.org/). GSE112791 (GPL570 platform) and GSE101685 datasets was selected from the Gene Expression Omnibus (GEO) database (16) (http://www.ncbi.nlm.nih.gov/geo/), and none of the selected tissue samples had treatment scenarios. Differential expression of *MPP6* mRNA in normal liver and HCC tissues were compared by the GEO2R online tool (17). R software was applied for integrating, analyzing and visualizing the data. Excel-VLOOKUP was employed to match and combine data.

### 2.2 Cell lines and cell culture

The human normal hepatic cell line LO2 was provided by Nanjing KeyGen Biotech. Co., Ltd. (Nanjing, China), and HCC cell lines (Huh7, Hep3B, BEL-7404 and SMMC-7721) were obtained from the Chinese Academy of Sciences (Shanghai, China). These cells were grown at 37°C in 5% CO_2_ in DMEM (Gibco, CA, USA) or RPMI 1640 medium (Gibco, CA, USA) containing 10% fetal bovine serum (Gibco, CA, USA).

### 2.3 qRT−PCR

TRIzol reagent (Invitrogen, CA, USA) was utilized to extract the entire RNA, and cDNA was generated using AMV reverse transcriptase (Promega, Wisconsin, USA). cDNA was amplified with RR820A SYBR^®^ Premix Ex Taq™ II (Tli RNaseH Plus) (TaKaRa, Osaka, Japan) and a 7500 Fast Real-Time PCR System (Applied Biosystems, MD, USA). The above operations were carried out according to their instructions. The procedures for PCR quantification were performed as previously described (18, 19). Internal reference was the GAPDH gene. The primers were listed in **Supplementary Table 1**.

### 2.4 Western blotting

The protocols for protein extraction were from the above HCC cell lines and the subsequent processes were mentioned in our previous work (18, 19). The following primary antibodies and concentrations were utilized: *MPP6* (1:4000; Proteintech, Wuhan, China) and GAPDH (1:10000; Proteintech, Wuhan, China). The GAPDH gene was employed to standardize gene and protein expression.

### 2.5 Clinical sample collection

Twenty pairs of human HCC and matched adjacent noncancerous tissue samples were obtained at the time of surgery between May 2022 and November 2022 at our institution. None of the HCC patients had systemic antitumor treatment before surgery.

Our institution’s Ethics Committee authorized this research.

### 2.6 Immunohistochemistry (IHC) staining

Rabbit anti-human *MPP6*, VEGFA and VEGFR2 polyclonal antibodies were provided from Proteintech (Wuhan, China), and anti-CD34 monoclonal antibody, anti-CD3+/CD4+/CD8+ T-cell monoclonal antibodies, secondary antibodies, and SP kits were purchased from Fuzhou Maishin Biotechnology (Fuzhou, China). These tissues were routinely dewaxed, hydrated, subjected to antigen repair with citrate buffer solution, and then assessed. All operating procedures strictly adhered to kit instructions. The *MPP6*, VEGFA and VEGFR2 primary antibody dilation ratio was 1:200, and anti-CD34 monoclonal antibody and anti-CD3+/CD4+/CD8+ T-cell monoclonal antibodies were ready-to-use antibodies. According to the positive signal, staining intensity was classified by unstained as negative, light yellow as (+), yellowish-brown as (++) and brown as (+++). Samples with MPP6 staining intensities of (+) and (+++) were chosen for VEGFA, VEGFR2 and CD34 staining to observe angiogenesis and CD3+/CD4+/CD8+ T-cell staining to evaluate immune cell distribution.

### 2.7 Analysis of *MPP6* expression and correlation with clinicopathological characteristics of HCC patients

*MPP6* expression in normal liver and HCC tissues was compared utilizing the R package “limma” in XIANTAO Academic (20) (https://www.xiantao.love/products) and the UALCAN website (21) (http://ualcan.path.uab.edu/). HCC samples were divided into high and low *MPP6* expression groups according to the median *MPP6* expression, and clinicopathological characteristics of HCC patients in different *MPP6* expression groups were evaluated by the R packages “ComplexHeatmap” and “ggpubr” in XIANTAO Academic.

### 2.8 Prognostic analysis of *MPP6* in HCC

The value of *MPP6* in predicting HCC patient survival was analyzed utilizing the log-rank test and Cox regression model based on the TCGA and ICGC (Japan cohort) databases.

### 2.9 Differentially expressed gene (DEG) enrichment analysis

The R package “DESeq2” was utilized to screen DEGs of high and low *MPP6* expression groups in TCGA database, and defined a subset of significantly DEGs according to |logFC|>1.5 and adjusted *P*<0.05 for GO analysis, which was performed by the Metascape online database (22) (https://metascape.org/gp/index.html#/main/step1). Gene set enrichment analysis (GSEA) in high and low *MPP6* expression groups was conducted by the R package “clusterProfiler” with the reference dataset from the MSigDB website (23) (https://www.gsea-msigdb.org/gsea/msigdb/index.jsp), and significant enrichment was defined as FDR<0.25, adjusted *P*<0.05, and normalized enrichment score (NSE) >1.

### 2.10 Correlation analysis of *MPP6* and angiogenesis

The CAMOIP online database (24) (http://www.camoip.net/) was used to investigate the connection between *MPP6* and angiogenesis. The correlation between the expression of *MPP6* and angiogenesis-related factors (VEGFA, VEGFR2 and CD34) was assessed by the GEPIA website (25) (http://gepia.cancer-pku.cn/) and validated by IHC staining in the study cohort.

### 2.11 Correlation between *MPP6* and the tumor microenvironment (TME)

The TISCH open access tool (26) (http://tisch.comp-genomics.org) was applied to assess the link between *MPP6* and TME features at the single-cell level, with screening parameters of Cell-type annotation: Cell type (major-lineage); Cancer type: LIHC (Liver Hepatocellular Carcinoma); Cell type included in datasets: No parameter set; Lineage for calculating correlation: All lineage; Treatment: No treatment; Primary/Metastatic: Primary. The “estimateScore” algorithm was employed to figure out the stromal score, immune score, ESTIMATE score, and tumor purity for all HCC samples, and these values across were compared among different *MPP6* expression groups. In addition, this study also examined the connection between the expression of *MPP6* and immune checkpoint genes.

Single-sample GSEA (ssGSEA) was carried out utilizing the R package “GSVA” to compare immune cell infiltration among different *MPP6* expression groups; the immune cell score for each HCC sample was determined to assess the association between *MPP6* and immune cell infiltration.

### 2.12 TMB analysis

TMB data was downloaded in TCGA database, and the R package “maftools” was applied to evaluate nucleotide compilation data, evaluate TMB for HCC patients with different *MPP6* expression levels, and examine how TMB affects HCC patient survival. HCC samples were divided into high and low TMB groups according to the optimal cutoff value of TMB. Considering that the high and low TMB groups both contained high (representing high risk) and low (representing low risk) *MPP6* expression samples, thus HCC samples could be divided into four groups: H-TMB+ high risk, H-TMB+ low risk, L-TMB+ high risk, and L-TMB+ low risk. The survival of HCC patients from these four groups were compared.

### 2.13 Comparison of treatment response under different expression levels of *MPP6*

Using the R package “pRRophetic”, we calculated and compared the half-maximal inhibitory concentration (IC50) values of several medications in HCC patients with different *MPP6* expression levels. We acquired the immunophenotype score (IPS) data of HCC samples from the TCIA online database (27) (https://www.cancerimagingarchive.net/), with *s*creening parameters: TCGA; LIHC; All genders; All stages; All T stages; All N stages; All M stages; All immune response, and evaluated the efficacy of immunotherapy based on IPS. IPS predicts patients’ response to immunotherapy based on immunogenicity, and it is widely assumed that high IPS indicates superior efficacy of immune checkpoint inhibitors (28).

### 2.14 Statistical analysis

R software (version 3.6.3) and GraphPad Prism 8.0 were employed to analyze data. A t test, or Wilcoxon test was applied to compare two independent groups. The Kruskal-Wallis test or ANOVA test was conducted to examine over two groups. The χ^2^ test was utilized to compare rates or percentages. Log-rank tests and Cox regression models were employed to survival analysis, and Pearson and Spearman correlation analyses were used for continuous factors and hierarchical factors, respectively. *P*<0.05 was deemed statistically significant.

## 3 Results

### 3.1 *MPP6* expression in HCC

**Figure 1:**
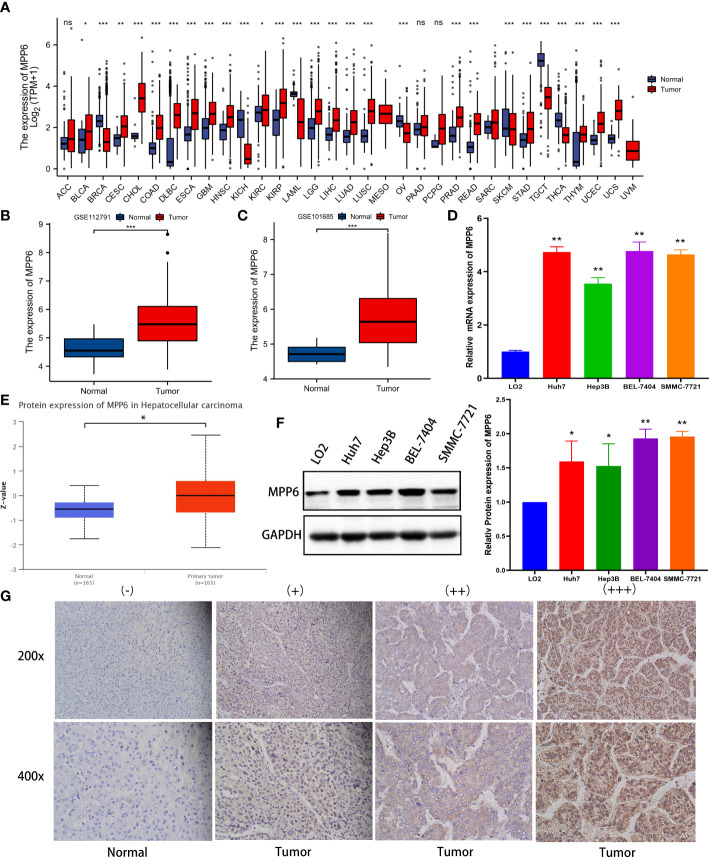
*MPP6* expression in HCC. **(A)** *MPP6* mRNA expression in malignant tumor and normal tissues based on the TCGA database. **(B, C)** *MPP6* mRNA expression in normal liver and HCC tissues based on GSE112791 (GPL570 platform) and GSE101685 datasets. **(D)** *MPP6* mRNA expression in HCC cell lines (Huh7, Hep3B, BEL-7404, and SMMC-7721) compared with the normal hepatic cell line LO2 by qRT−PCR. **(E)** *MPP6* protein expression in normal liver and HCC tissues based on the UALCAN website. **(F)** *MPP6* protein expression in LO2 and HCC cell lines (Huh7, Hep3B, BEL-7404, and SMMC-7721) by Western blotting. **(G)** Immunohistochemical staining analysis of *MPP6* in normal liver and HCC tissues. *P <0.05; **P <0.01; ***P <0.001; ns: *P >*0.05.

In the TCGA database, *MPP6* mRNA was markedly overexpressed in various malignant tumor tissues, including HCC tissues (*P* < 0.05, **Figure 1A**). GSE112791 and GSE101685 datasets analyses also revealed increased *MPP6* mRNA expression in HCC tissues (all *P* < 0.001, **Figures 1B, C**). qRT−PCR further verified that *MPP6* mRNA was expressed at higher levels in HCC cell lines such as Huh7, Hep3B, BEL-7404 and SMMC-7721 than in the normal liver cell line LO2 (all *P* < 0.05, **Figure 1D**). At the protein level, *MPP6* expression was notably higher in HCC tissues than in normal liver tissues from the CPTAC online database (21) (*P*<0.05, **Figure 1E**), and this result was confirmed in HCC cell lines and tissues by Western blotting (all *P*<0.05, **Figure 1F**) and IHC staining (**Figure 1G**), respectively.

### 3.2 Clinicopathological characteristics analysis

**Figure 2:**
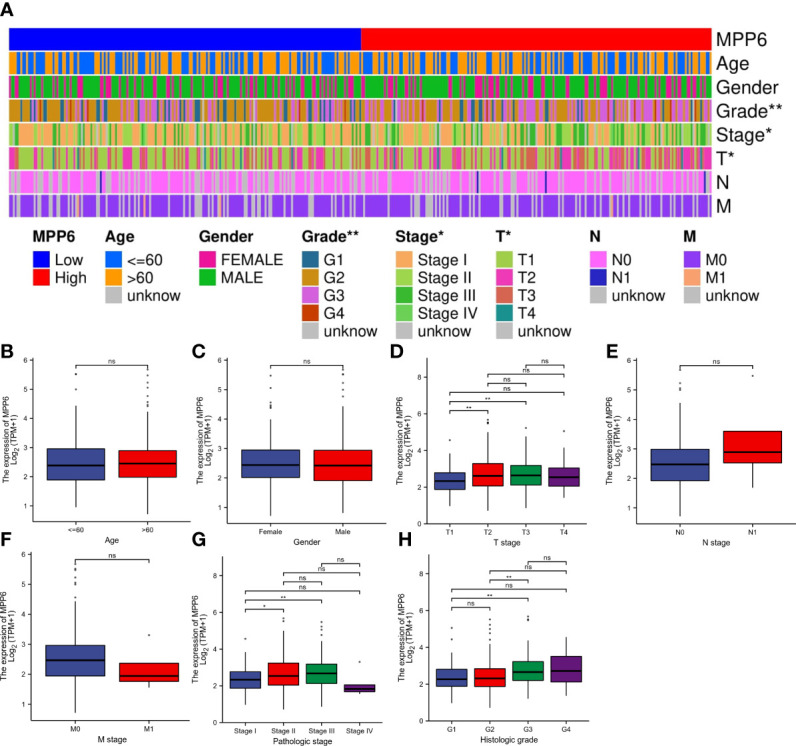
Correlation analysis of *MPP6* expression with clinicopathological characteristics in HCC patients based on the TCGA database. **(A)** Heatmap showing the connection between *MPP6* and clinicopathological characteristics in HCC. **(B–H)** Relationship between *MPP6* and age, gender, T stage, N stage, M stage, pathologic stage, and histologic grade. *P <0.05; **P <0.01; ns, *P >*0.05.

In the TCGA database, *MPP6* expression had a positive relation to the T stage, pathologic stage, and histologic grade of HCC patients (all *P* < 0.05, **Figure 2**; **Supplementary Tables 2, 3**).

### 3.3 Prognostic potential of *MPP6* in HCC

**Figure 3:**
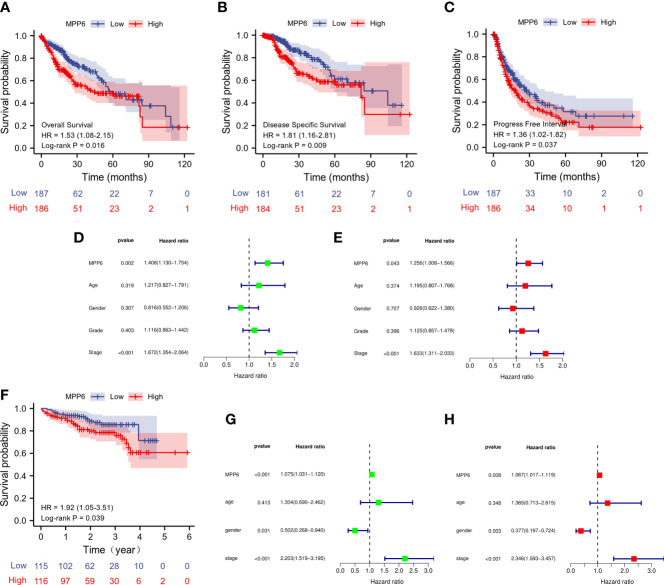
Prognostic analysis of *MPP6* expression in HCC based on TCGA and ICGC databases. **(A–C)** KM curves of OS, DSS, and PFI for HCC patients with different *MPP6* expression in the TCGA database. **(D, E)** Cox regression analyses of OS in the TCGA database. **(F)** KM curves of OS for HCC patients with different *MPP6* expression in Japan cohort released in the ICGC database. **(G, H)** Cox regression analyses of OS in Japan cohort released in the ICGC database.

In the TCGA database, high *MPP6* expression suggested shorter overall survival (OS), disease-specific survival and progression-free interval than low *MPP6* expression in HCC patients (all *P*<0.05, **Figures 3A–C**), and the Cox regression model revealed that *MPP6* expression and pathologic stage were independent predictors of OS (all *P*<0.05, **Figures 3D, E**). **Figure 3F** shows that high *MPP6* expression suffered worse OS than low *MPP6* expression in HCC patients based on the ICGC cohort (*P*=0.039), and the Cox regression analysis revealed that *MPP6* can independently predict HCC patient OS (all *P*<0.05, **Figures 3G, H**). **Supplementary Table 4** displays HCC patient information in the ICGC database.

### 3.4 *MPP6* related DEGs analysis and its relationship with angiogenesis

**Figure 4:**
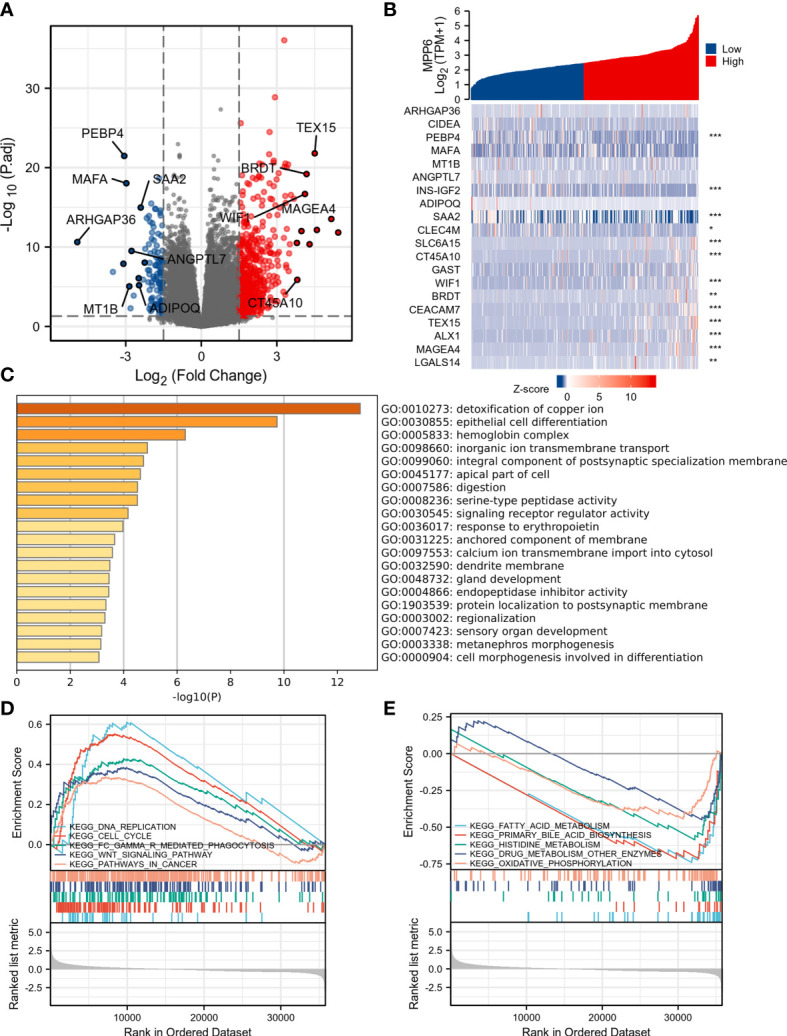
DEGs analysis of high and low *MPP6* expression groups in TCGA database. **(A)** Volcano plot of DEGs of high and low *MPP6* expression groups. **(B)** Heatmap of the top 20 DEGs correlated with *MPP6.* **(C)** GO enrichment analysis based on significantly DEGs correlated with *MPP6*. **(D)** Identification of *MPP6*-related signaling pathways by GSEA in high *MPP6* expression groups. **(E)** Identification of *MPP6*-related signaling pathways by GSEA in low *MPP6* expression groups.

A total of 519 DEGs in TCGA database were discovered, and a volcano map was generated (**Figure 4A**); the top 20 DEGs are demonstrated in **Figure 4B**. The GO results revealed that these DEGs were enriched in biological activities, mainly including detoxification of copper ion, epithelial cell differentiation, hemoglobin complex, and inorganic ion transmembrane transport (**Figure 4C**). KEGG pathway analysis in GSEA demonstrated that in the high *MPP6* expression group, the main enriched pathways were DNA replication, cell cycle, Fc gamma γ mediated phagocytosis, WNT signaling pathway, and cancer-related signaling pathways, while in the low *MPP6* expression group, the main enriched pathways were fatty acid metabolism, primary bile acid biosynthesis, histidine metabolism, drug metabolism other enzymes, and oxidative phosphorylation-related pathways, which are demonstrated in **Figures 4D, E** and **Supplementary Table 5**.

**Figure 5:**
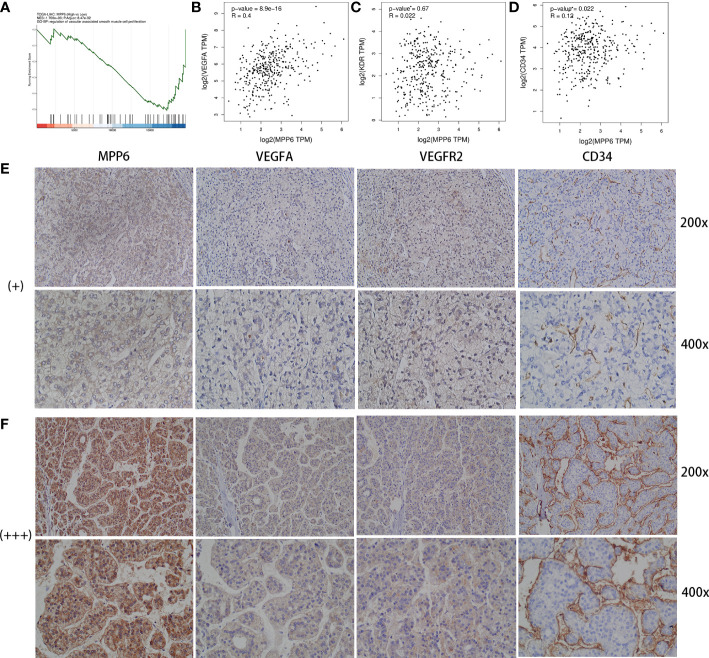
Analysis between *MPP6* expression and angiogenic factors in HCC. **(A)** GO enrichment analysis by CAMOIP online database. **(B–D)** Relevance of expression between *MPP6* and VEGFA, VEGFR2 and CD34 based on the GEPIA database. **(E, F)** Expression of VEGFA, VEGFR2 and CD34 in HCC samples with *MPP6* staining intensity of “+” and “+++” based on the study cohort.

GSEA by CAMOIP online database found that *MPP6* could inhibit the proliferation of vascular-associated smooth muscle cells (**Figure 5A**). The GEPIA online database indicated that *MPP6* expression had a positive correlation with the expression levels of VEGFA (r=0.4, *P*=8.9e-16, **Figure 5B**) and CD34 (r=0.12, *P*=0.022, **Figure 5D**), while no significant correlation was observed with VEGFR2 (r=0.022, *P*=0.67, **Figure 5C**). The expression of VEGFA, VEGFR2 and CD34 were positively linked with *MPP6* expression by IHC staining in the study cohort (**Figures 5E, F**).

### 3.5 Correlation analysis of *MPP6* with TME

**Figure 6:**
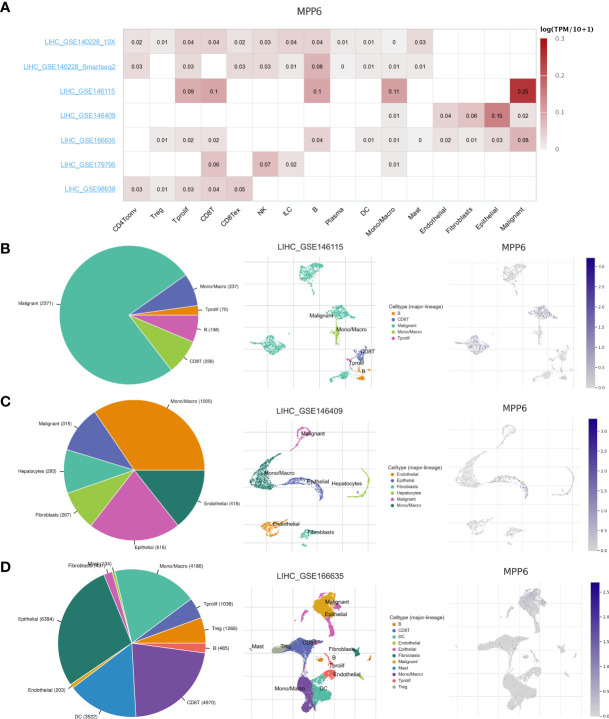
Correlation between *MPP6* and TME at the single-cell level in the TISCH open access tool. **(A)** Heatmap showing *MPP6* expression in various cells from diverse datasets. **(B–D)** *MPP6* expression in various cells based on the GSE146115, GSE146409 and GSE166635 cohorts.

Single-cell analysis revealed that a total of seven HCC datasets in this study. The findings indicated that *MPP6* was expressed at different levels in malignant cells, stromal cells, and immune cells (**Figure 6A**). In the LIHC-GSE146115 dataset, *MPP6* was expressed in malignant cells, T cells, B cells and macrophages, and *MPP6* expression was highest in malignant cells (**Figure 6B**). In the LIHC-GSE146409 dataset, *MPP6* was expressed at different levels in macrophages, epithelial cells, endothelial cells, malignant cells, hepatocytes, and fibroblasts, and *MPP6* expression was highest in macrophages (**Figure 6C**). In the LIHC-GSE166635 dataset, *MPP6* was mostly expressed in epithelial cells, CD8+ T cells and macrophage cells (**Figure 6D**).

**Figure 7:**
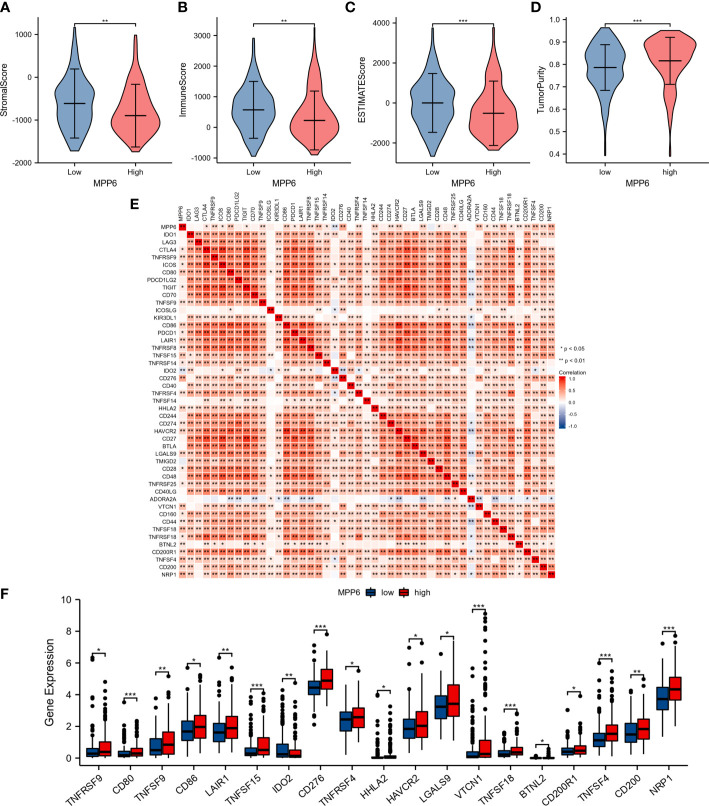
Relationship between *MPP6* and alteration of the immune landscape. **(A–D)** Comparison of stromal score, immune score, ESTIMATE score and tumor purity in different *MPP6* expression groups. **(E)** Relevance of expression between *MPP6* and immune checkpoint genes. **(F)** Differential expression of immune checkpoint genes in different *MPP6* expression groups. *P <0.05; **P <0.01; ***P <0.001.

Further analysis revealed that, compared to the high *MPP6* expression group, the low *MPP6* expression group had markedly higher stromal score, immune score, and ESTIMATE score, whereas tumor purity in the low *MPP6* expression group was lower (all *P*<0.05, **Figures 7A–D**). In addition, *MPP6* was correlated with various immune checkpoint genes (*P*<0.05, **Figure 7E**). There were notable differences in *TNFRSF9*, *CD80*, *TNFSF9*, *CD86*, *LAIR1*, *TNFSF15*, *IDO2*, *CD276*, *TNFRSF4*, *HHLA2*, *HAVCR2*, *LGALS9*, *VTCN1*, *TNFSF18*, *BTNL2*, *CD200R1*, *TNFSF4*, *CD200*, and *NRP1* expression in groups with different *MPP6* expression (all *P* < 0.05, **Figure 7F**).

**Figure 8:**
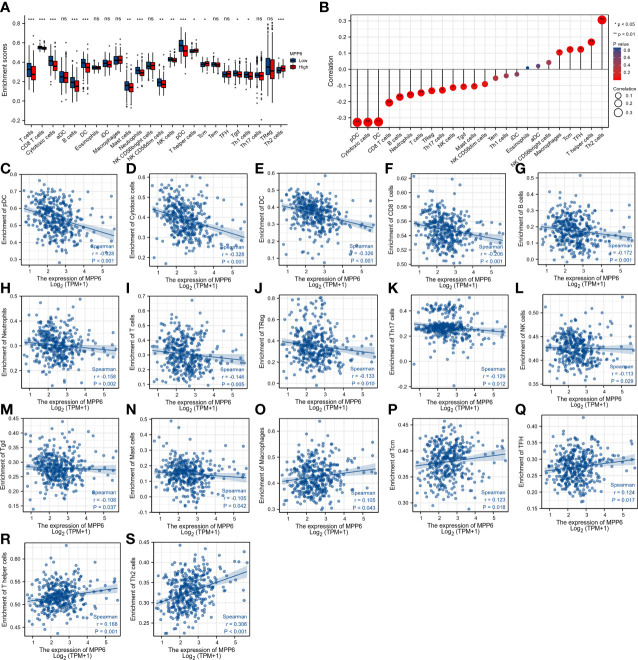
Correlation analysis of *MPP6* with immune cell infiltration in HCC. **(A)** Differences in immune cell infiltration in different *MPP6* expression groups. **(B)** Relationship between *MPP6* and immune cell infiltration. **(C–S)** Correlation of *MPP6* expression with infiltration level of pDC, cytotoxic cells, DC, CD8 T cells, B cells, neutrophils, T cells, Treg, Th17 cells, NK cells, Tgd, mast cells, macrophages, Tcm, TFH, T helper cells, and Th2 cells. *P <0.05; **P <0.01; ***P <0.001; ns, *P >*0.05.

T cells, CD8 T cells, cytotoxic cells, B cells, dendritic cells (DCs), mast cells, neutrophils, NK CD56dim cells, NK cells, pDCs, Tgd cells, and Th1 cells had higher infiltration levels in the low *MPP6* expression group, while the infiltration levels of T helper cells, Tcm cells, and Th2 cells were higher in the high *MPP6* expression group (all *P* < 0.05, **Figure 8A**). Further analysis demonstrated that *MPP6* was related to various immune cells (**Figure 8B**), such as pDCs, cytotoxic cells, DCs, CD8 T cells, B cells, neutrophils, T cells, Tregs, Th17 cells, NK cells, Tgd cells, mast cells, macrophages, Tcm cells, TFH, T helper cells, and Th2 cells (all *P*<0.05, **Figures 8C–S**).

**Figure 9:**
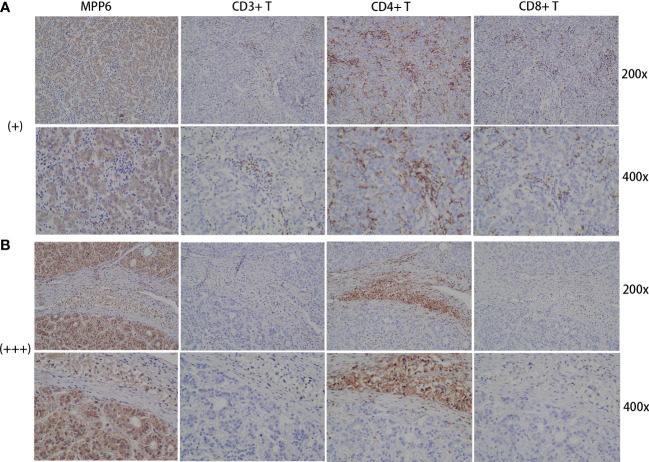
Association between immune evasion and *MPP6* expression. **(A)** Distribution of CD3+ T cells, CD4+ T cells and CD8+ T cells in HCC samples with *MPP6* staining intensity of “+” based on the study cohort. **(B)** Distribution of CD3+ T cells, CD4+ T cells and CD8+ T cells in HCC samples with *MPP6* staining intensity of “+++” based on the study cohort.

In the study cohort, IHC staining revealed that CD3+ T cells, CD4+ T cells, and CD8+ T cells were diffusely distributed in the HCC tumor parenchyma when *MPP6* expression was “+” (**Figure 9A**). In contrast, when *MPP6* expression was “+++”, CD3+ T cells, CD4+ T cells and CD8+ T cells were mainly concentrated in the peritumoral stroma of HCC samples, with few or no T cells penetrating into the tumor parenchyma (**Figure 9B**).

### 3.6 Association between *MPP6* expression and TMB

*MPP6* expression was positively associated with TMB (**Supplementary Figures 1A, B**). HCC patients with high TMB exhibited worse survival (*P*<0.05, **Supplementary Figure 1C**). The worst survival was found in HCC patients with both high *MPP6* expression and high TMB (*P*<0.05, **Supplementary Figure 1D**).

### 3.7 Prediction of sensitivity to different kinds of drugs and potential small-molecule inhibitors

**Figure 10:**
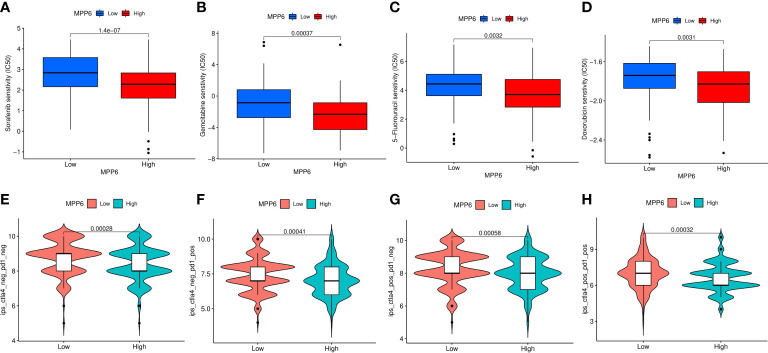
Prediction of treatment responses of HCC patients. **(A–D)** The IC50 values of sorafenib, gemcitabine, 5-FU and doxorubicin in different *MPP6* expression groups. **(E–H)** Response to immunotherapy in high and low *MPP6* expression patients.

Finally, we compared the drug sensitivity of HCC patients in different *MPP6* expression groups. Compared to HCC patients in the low *MPP6* expression group, the IC50 values of sorafenib, gemcitabine, 5-FU and doxorubicin were lower for HCC patients with high *MPP6* expression (all *P*<0.05, **Figures 10A–D**), which means that high *MPP6* expression patients were more responsive to sorafenib, gemcitabine, 5-FU and doxorubicin. In contrast, for HCC patients who received immune checkpoint inhibitor therapy, the high *MPP6* group had a higher IPS than the low *MPP6* group (all *P*<0.05, **Figures 10E–H**).

## 4 Discussion

*MPP6* was demonstrated to be significantly overexpressed in HCC tissues by bioinformatics analyses of several public databases, and this finding was further validated by qRT−PCR, Western blotting and IHC analyses using HCC cell lines and tissues of the study cohort. Clinicopathological characteristic analyses demonstrated that *MPP6* expression was positively related to T stage, pathological stage and histological grade. High *MPP6* expression patients exhibited adverse survival. These findings suggested that *MPP6* was related to the malignant phenotype of HCC patients and has the potential to predict HCC patient prognosis.

GO biological processes enriched in DEGs mainly included the detoxification of copper ion. Studies have demonstrated that tumorigenesis is correlated with copper metabolism (29, 30). GSEA revealed that in the high *MPP6* expression group, the main enriched pathways were the synthesis of genetic material, cell cycle, WNT signaling pathway and cancer-related pathways. Uncontrolled cell growth and hyperproliferation are the characteristics of malignant tumors (31, 32). Cell cycle disorder and increased abnormal synthesis of genetic material also support the association between *MPP6* expression and the malignant phenotype of HCC patients. Studies, including ours, have proven that the WNT signaling pathway is engaged in HCC progression (33, 34). It has been shown in several studies that the WNT signaling pathway is closely connected with immune evasion (35, 36). Qu et al. (37) also revealed that the WNT signaling pathway can promote angiogenesis by regulating the expression of angiogenesis-related factors, which contributes to HCC metastasis. These events may be involved in the acquisition of the HCC malignant phenotype in the high *MPP6* expression group. However, in the low *MPP6* expression group, metabolism-related pathways were mainly enriched. To meet the energy needs for HCC cell proliferation, the metabolic balance of the entire organism is remodeled, which promotes cancer cell growth and migration (38, 39).

For advanced HCC patients, immunotherapy combined with antiangiogenic therapy is the standard first-line therapy, and the GSEA results suggest that the role of *MPP6* in HCC angiogenesis and immune evasion is worth further exploration. GSEA revealed that *MPP6* expression is inversely correlated with the proliferation of vascular-associated smooth muscle cells. However, most tumor vessels lack smooth muscle layers, which facilitates tumor cell invasion and migration (40). Bioinformatics and IHC staining jointly confirmed that *MPP6* expression was positively related to the expression of angiogenesis-related factors (VEGFA and CD34). Based on the above findings, we speculated that *MPP6* may promote HCC angiogenesis, which contributes to HCC cell invasion and migration and affects HCC patient prognosis (41–43).

This study indicated that *MPP6* can be expressed in malignant cells and other cells, including immune cells and stromal cells, suggesting that *MPP6* is closely associated with the TME and may induce an effect through a variety of cells. In addition, compared to the high *MPP6* expression group, the low *MPP6* expression group had a higher infiltration level of stromal cells and immune cells and a lower percentage of tumor cells. The above results were also conformity to the results of the immune cell analysis; that is, *MPP6* expression was inversely related to most immune cells infiltration and positively related to a few immune cells infiltration. DCs, T cells, B cells, neutrophils, and NK cells can identify and kill tumor cells in a specific or nonspecific way, which can inhibit tumor growth, migration and invasion, thereby improving HCC patient prognosis (44–46). Macrophages are innate immune cells. Macrophages in the TME are called tumor-associated macrophages (TAMs). TAMs are vital for the immune response against tumors and may affect immunotherapy efficacy. For example, it has been shown that TAMs can mediate resistance to immune checkpoint inhibitors by regulating T-cell apoptosis and proliferation (47, 48). In a subsequent study evaluating immunotherapy efficacy, we found that patients with low *MPP6* expression responded well to immunotherapy. All these results are consistent.

For patients with cancer, the immune system mobilizes lymphocytes to attack tumor cells, but the lymphocytes that truly work in the antitumor immune response are those that penetrate deeply into the tumor parenchyma, and the number of tumor-infiltrating lymphocytes (TILs) directly affects patient prognosis (49, 50). TILs are lymphocytes isolated from tumor tissues and mainly include CD3+ T cells, CD4+ T cells, and CD8+ T cells, especially CD8+ T cells, which are the basis for producing an effect of immunotherapy (51). The number of TILs has increased in patients who respond well to immunotherapy (52, 53). Immune evasion refers to tumors that manage to escape being identified and killed by the immune system. One of the hallmarks of the TME in patients with immune evasion is the reduced number of TILs, which means that lymphocytes can barely penetrate the tumor stroma and enter the tumor parenchyma to work (54). Therefore, it is reasonable to hypothesize that elevated *MPP6* expression mediates immune evasion in HCC patients, and this speculation was supported by a subsequent study that discovered that patients with low *MPP6* expression respond well to immunotherapy.

TMB is an indicator used to evaluate the frequency of gene mutation. This study found that *MPP6* expression was positively related to TMB, while HCC patients with high TMB exhibited poor prognosis. It is speculated that high TMB might lead to impaired immune cell function by causing continuous antigen exposure (55), which also provides an indirect explanation of why high *MPP6* expression patients have an adverse survival. In the subsequent analysis of treatment response, we also analyzed the IC50 of sorafenib, gemcitabine, 5-FU and doxorubicin and found that high *MPP6* expression patients respond better to these drugs than low *MPP6* expression patients. We speculated that this may be related to the higher frequency of gene mutations, higher degree malignancy and increased number of proliferative cells in these patients. Additionally, there were notable differences in the expression of various immune checkpoint genes in groups with different *MPP6* expression, indicating that *MPP6* may contribute to the selection of immunotherapeutic targets for HCC.

In summary, this study demonstrates that elevated *MPP6* expression correlates with unfavorable clinicopathological features and an adverse survival in HCC patients. *MPP6* is related to angiogenesis induction and immune evasion and could also be used in assessing TMB and treatment response; thus, *MPP6* might serve as a novel prognostic predictor or potential therapeutic target for HCC.

## Data availability statement

The original contributions presented in the study are included in the article/**Supplementary Material**. Further inquiries can be directed to the corresponding author.

## Ethics statement

The studies involving human participants were reviewed and approved by Ethics Committee of The First Affiliated Hospital of Bengbu Medical College. The patients/participants provided their written informed consent to participate in this study.

## Author contributions

QC and WW contributed equally as co-first authors in conducting main experiments, performing data analysis, and drafting the manuscript. JL assisted with data analysis and manuscript preparation. ZL and WJ contributed to material preparation and experiments performance. JY and WZ participated in data collection and discussion. YY conceived the study and helped to revise the manuscript. All authors contributed to the article and approved the submitted version.

## Supplementary material

Supplementary Figure 1 Correlation between TMB and *MPP6* expression, and their relationship with survival of HCC patients in the TCGA database. **(A, B)** Oncoplots of the top 15 mutated genes in different *MPP6* expression groups. **(C)** KM curves of different TMB groups. **(D)** The effect of *MPP6* expression combined with TMB on HCC patient prognosis.
