# Supplementary figures and images for: Elevated MPP6 expression correlates with an unfavorable prognosis, angiogenesis and immune evasion in hepatocellular carcinoma
Source: Front Immunol. 2023 May 3;14:1173848. doi: 10.3389/fimmu.2023.1173848 (PMC10189050; doi:10.3389/fimmu.2023.1173848)

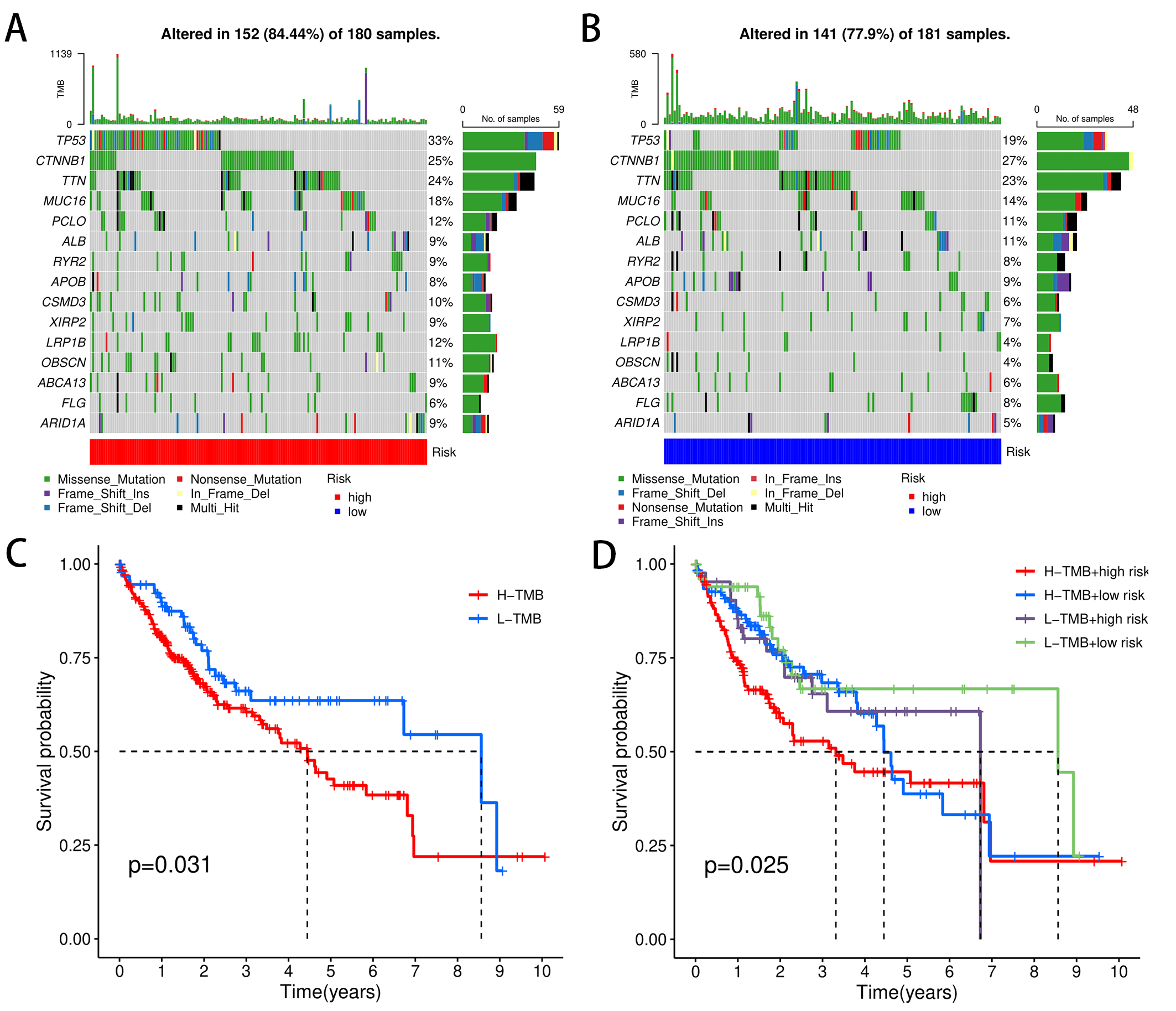

Supplement: Supplementary Figure 1 — Correlation between TMB and MPP6 expression, and their relationship with survival of HCC patients in the TCGA database. (A, B) Oncoplots of the top 15 mutated genes in different MPP6 expression groups. (C) KM curves of different TMB groups. (D) The effect of MPP6 expression combined with TMB on HCC patient prognosis. [file Image_1.tif]
