# Supplementary material for: Elevated MPP6 expression correlates with an unfavorable prognosis, angiogenesis and immune evasion in hepatocellular carcinoma
Source: Front Immunol. 2023 May 3;14:1173848. doi: 10.3389/fimmu.2023.1173848 (PMC10189050; doi:10.3389/fimmu.2023.1173848)
Supplement: Supplementary file 2 [file Table_1.docx]

**Supplementary Table 1** The primers used for qRT‒PCR analysis

| Gene | Forword (5’-3’) | Reverse (5’-3’) | Product size (bps) |
| --- | --- | --- | --- |
| MPP6 | CTGGACGGACTTGCATTCTG | AGTGATTCCTGCATCCACCA | 149 |
| GAPDH | TGAACGGGAAGCTCACTG | GCTTCACCACCTTCTTGATG | 120 |
