# Supplementary material for: Elevated MPP6 expression correlates with an unfavorable prognosis, angiogenesis and immune evasion in hepatocellular carcinoma
Source: Front Immunol. 2023 May 3;14:1173848. doi: 10.3389/fimmu.2023.1173848 (PMC10189050; doi:10.3389/fimmu.2023.1173848)
Supplement: Supplementary file 3 [file Table_2.docx]

**Supplementary Table 2** Clinicopathological characteristics of HCC patients with different MPP6 expression in TCGA database.

| Characteristic | Low-MPP6 | High-MPP6 | *P* |
| --- | --- | --- | --- |
| Age, n (%) |  |  |  |
| <=60 | 91 (24.4%) | 86 (23.1%) | 0.715 |
| >60 | 96 (25.7%) | 100 (26.8%) |  |
| Gender, n (%) |  |  |  |
| Female | 59 (15.8%) | 62 (16.6%) | 0.825 |
| Male | 128 (34.2%) | 125 (33.4%) |  |
| T stage, n (%) |  |  |  |
| T1 | 105 (28.3%) | 78 (21%) | 0.031* |
| T2 | 40 (10.8%) | 55 (14.8%) |  |
| T3 | 33 (8.9%) | 47 (12.7%) |  |
| T4 | 6 (1.6%) | 7 (1.9%) |  |
| N stage, n (%) |  |  |  |
| N0 | 123 (47.7%) | 131 (50.8%) | 0.623 |
| N1 | 1 (0.4%) | 3 (1.2%) |  |
| M stage, n (%) |  |  |  |
| M0 | 129 (47.4%) | 139 (51.1%) | 0.358 |
| M1 | 3 (1.1%) | 1 (0.4%) |  |
| Pathologic stage, n (%) |  |  |  |
| Stage I | 101 (28.9%) | 72 (20.6%) | 0.010** |
| Stage II | 39 (11.1%) | 48 (13.7%) |  |
| Stage III | 34 (9.7%) | 51 (14.6%) |  |
| Stage IV | 4 (1.1%) | 1 (0.3%) |  |
| Histologic grade, n (%) |  |  |  |
| G1 | 36 (9.8%) | 19 (5.1%) | 0.005** |
| G2 | 96 (26%) | 82 (22.2%) |  |
| G3 | 48 (13%) | 76 (20.6%) |  |
| G4 | 5 (1.4%) | 7 (1.9%) |  |

* *P* <0.05; ** *P* <0.01; *** *P* <0.001.
