# Supplementary material for: Elevated MPP6 expression correlates with an unfavorable prognosis, angiogenesis and immune evasion in hepatocellular carcinoma
Source: Front Immunol. 2023 May 3;14:1173848. doi: 10.3389/fimmu.2023.1173848 (PMC10189050; doi:10.3389/fimmu.2023.1173848)
Supplement: Supplementary file 4 [file Table_3.docx]

**Supplementary Table 3** Logistic regression analysis of MPP6 expression and clinicopathological characteristics of HCC patients in TCGA database.

| Characteristic | Total (N) | Odds Ratio (OR) | *P* |
| --- | --- | --- | --- |
| Age (>60 vs. <=60) | 373 | 1.151 (0.766-1.730) | 0.499 |
| Gender (Male vs. Female) | 374 | 0.885 (0.573-1.365) | 0.581 |
| T stage (T2&T3&T4 vs. T1) | 371 | 1.777 (1.180-2.688) | 0.006** |
| N stage (N1 vs. N0) | 258 | 2.773 (0.350-56.463) | 0.380 |
| M stage (M1 vs. M0) | 272 | 0.309 (0.015-2.450) | 0.312 |
| Pathologic stage (Stage II& Stage III& Stage IV vs. Stage I) | 350 | 1.739 (1.141-2.661) | 0.010** |
| Histologic grade (G3&G4 vs. G1&G2) | 369 | 2.047 (1.334-3.162) | 0.001*** |

* *P* <0.05; ** *P* <0.01; *** *P* <0.001.
