# Supplementary material for: Elevated MPP6 expression correlates with an unfavorable prognosis, angiogenesis and immune evasion in hepatocellular carcinoma
Source: Front Immunol. 2023 May 3;14:1173848. doi: 10.3389/fimmu.2023.1173848 (PMC10189050; doi:10.3389/fimmu.2023.1173848)
Supplement: Supplementary file 5 [file Table_4.docx]

**Supplementary Table 4** Clinicopathological characteristics of HCC patients with different MPP6 expression in ICGC database.

| Characteristic | Low-MPP6 | High-MPP6 | *P* |
| --- | --- | --- | --- |
| Age, n (%) |  |  |  |
| <=60 | 30 (13%) | 19 (8.2%) | 0.115 |
| >60 | 86 (37.2%) | 96 (41.6%) |  |
| Gender, n (%) |  |  |  |
| Female | 25 (10.8%) | 36 (15.6%) | 0.126 |
| Male | 91 (39.4%) | 79 (34.2%) |  |
| Pathologic stage, n (%) |  |  |  |
| Stage I | 26 (11.3%) | 10 (4.3%) | 0.027* |
| Stage II | 51 (22.1%) | 54 (23.4%) |  |
| Stage III | 32 (13.9%) | 39 (16.9%) |  |
| Stage IV | 7 (3%) | 12 (5.2%) |  |

* *P* <0.05; ** *P* <0.01; *** *P* <0.001.
