# Supplementary material for: Elevated MPP6 expression correlates with an unfavorable prognosis, angiogenesis and immune evasion in hepatocellular carcinoma
Source: Front Immunol. 2023 May 3;14:1173848. doi: 10.3389/fimmu.2023.1173848 (PMC10189050; doi:10.3389/fimmu.2023.1173848)
Supplement: Supplementary file 6 [file Table_5.docx]

**Supplementary Table 5** Signaling pathways correlated with MPP6 expression.

| ID | NES | *P.* adjusted |
| --- | --- | --- |
| KEGG_PATHWAYS_IN_CANCER | 1.603883 | 0.024405 |
| KEGG_CELL_CYCLE | 2.368796 | 0.024405 |
| KEGG_WNT_SIGNALING_PATHWAY | 1.706657 | 0.024405 |
| KEGG_DNA_REPLICATION | 2.096907 | 0.024405 |
| KEGG_FC_GAMMA_R_MEDIATED_PHAGOCYTOSIS | 1.762998 | 0.03353 |
| KEGG_PRIMARY_BILE_ACID_BIOSYNTHESIS | -2.09824 | 0.024405 |
| KEGG_FATTY_ACID_METABOLISM | -2.88621 | 0.024405 |
| KEGG_OXIDATIVE_PHOSPHORYLATION | -2.1118 | 0.024405 |
| KEGG_VALINE_LEUCINE_AND_ISOLEUCINE_DEGRADATION | -2.17222 | 0.024405 |
| KEGG_PEROXISOME | -2.71665 | 0.024405 |
